# Menstrual blood-derived stem cells and its mitochondrial treatment improve the ovarian condition of aged mice

**DOI:** 10.18632/aging.204043

**Published:** 2022-05-03

**Authors:** Qi Zhang, Chunlei Liu, Ling Yu, Xiaona Wang, Jianxiu Hao

**Affiliations:** 1 Medical School of Chinese PLA, Department of Obstetrics and Gynecology, The First Medical Center of PLA General Hospital, Beijing 100853, China; 2 Department of Transformation Medicine Center, The Medical Innovation Research Division of Chinese PLA General Hospital, Beijing 100853, China; 3 Senior Department of Obstetrics and Gynecology, The Seventh Medical Center of PLA General Hospital, Beijing 100853, China; 4 Key Laboratory of RNA Biology, Center for Big Data Research in Health, Institute of Biophysics, Chinese Academy of Sciences, Beijing 100101, China; 5 Department of Clinical Biobank Center, The Medical Innovation Research Division of PLA General Hospital, Beijing 100853, China

**Keywords:** MenSCs, mitochondria, 3D alginate gel, ovaries

## Abstract

Aging causes a decline in ovarian function and may contribute to ovarian failure and infertility. We investigated the effect of menstrual blood-derived mesenchymal stem cells (MenSCs) and their mitochondria on ovarian function in aged mice. We performed two treatment protocols: i) ovaries of recipient aged mice were treated *in vivo* with MenSCs 3D alginate gel; ii) ovaries were injected with mitochondria suspension and then incubated with mitochondrial 3D gel. Seven days after treatment, ovaries were harvested for histological assessment by HE staining and transcriptomic analysis by RNA-seq. Our data showed that after incubation with stem cell 3D gel, the MenSCs could be detected in the recipient mouse ovary. HE staining showed that the follicular state of aging ovary improved with both treatments. RNA-seq analysis showed that mitochondrial pathway-related genes were upregulated and significantly enriched in the ovaries treated by MenSCs or their mitochondria.

Conclusions: Treatment with MenSCs or their mitochondria can enhance the expression of mitochondrial pathway-related genes and promote the recovery of ovarian function in aged mice.

## INTRODUCTION

Ovary, the center of female fertility regulation, is one of the most rapidly aging organs [1, 2]. Aging is accompanied with a decrease in the number and quality of follicles in the ovary; however, the mechanism is still unclear. It may be associated with the acceleration of follicular atresia and the shrinking of the primordial follicle pool [3, 4]. Decreased ovarian reserve (DOR) refers to the decrease in the number of recruitable follicles in the ovaries and decreased quality of the eggs. DOR leads to insufficient secretion or absence of sex hormones and decreased fertility, which can progressively worsen into ovarian failure [5]. Clinically, DOR is characterized by a decrease in anti-Mullerian hormone (AMH) levels, the number of antral follicles (AFC), and elevated basic follicle-stimulating hormone (FSH) levels [6]. The incidence and development of DOR accelerates significantly with aging. Currently, ovarian function is restored mainly through hormone therapy and immunotherapy. However, the effects of these treatments are transient and one-off and cannot effectively promote the regeneration and repair of self-damaged ovarian tissue. In view of these limitations of conventional treatment methods, scientists are actively looking for new and effective alternatives [7, 8].

Stem cells have been studied for the treatment of premature ovarian failure (POF) [9, 10]. Mesenchymal stem cells (MSCs) treatment technology is an important branch of stem cell therapy, which is considered to be a new hope for organ damage repair in the future [11]. Menstrual blood-derived mesenchymal stem cells (MenSCs) are a recently discovered type of pluripotent stem cells, that reside the endometrium and continuously renew themselves with the menstrual cycle. MenSCs can be isolated directly from menstrual blood in a non-invasive way, avoiding ethical disputes, and thus constitute an easily accessible source of stem cells, that is renewed periodically [12, 13]. MenSCs highly express mesenchymal stem cell surface markers, exhibit stronger proliferative ability than bone marrow derived stem cells and have been confirmed to have multidirectional differentiation potential [13]. MenSCs can be induced to differentiate into cell lineages such as chondrocytes, adipocytes, osteoblasts, neural cell lines, cardiomyocytes, while maintaining high proliferative ability even after continuous passages for multiple generations. They also express and secrete a variety of cytokines such as VEGF, FGF, KGF and HGF; at the same time, exhibit low immunity, tumorigenicity, and immunosuppressive characteristics [14, 15].

The mitochondria, where cells carry out aerobic respiration, are associated with age-related decrease in female reproductive outcome [16, 17]. Ovarian granulosa cells are rich in mitochondria, which metabolize cytoplasmic glucose into pyruvate and transport it to oocytes to produce ATP and maintain their development [18]. The morphology of mitochondria in granulosa cells changes with age, accompanied by a significant decrease in the mitochondrial membrane potential and ATP synthesis. Apoptosis of granulosa cells increases with age, which may affect oocyte function or cause changes in the ovarian reserve [19].

In this study, we prepared and used mitochondrial suspension, mitochondrial 3D gel, and stem cell 3D gel derived from mouse MenSCs, to treat the ovaries of aged mice. We examined the availability and feasibility of MenSCs and their mitochondria in the treatment of ovarian aging. We further explored the possible mechanism by which MenSCs and their mitochondria repair ovarian dysfunction.

## RESULTS

### Macroscopic ovarian sizes of the recipient mice treated by MenSCs and mitochondria

**Figure 1:**
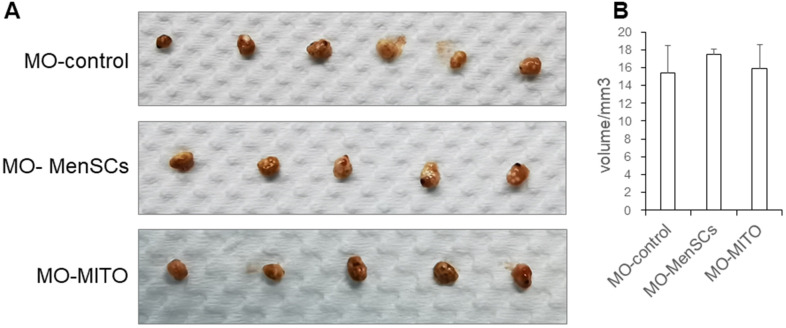
Macroscopic ovarian sizes (**A**) and representative bar graph summarizing the ovarian volumes (**B**) in the three groups after seven days of treatment.

Ovaries of aged recipient CD-1 (ICR) mice (10–12 months old) were treated with, either MenSCs 3D gel (MO-MenSCs) incubation or mitochondrial injection combined with mitochondrial 3D gel (MO-MITO) incubation. After 7 days following the treatment, ovaries were dissected from the recipient mice and their overall morphology and size was examined. The blank gel control group and MO-MITO treatment group had comparable ovarian volume. However, the MO-MenSCs treatment group had higher ovary size and volume compared to control group. (Figure 1A, 1B).

### Morphological changes in the recipient ovaries treated by MenSCs and mitochondria

**Figure 2:**
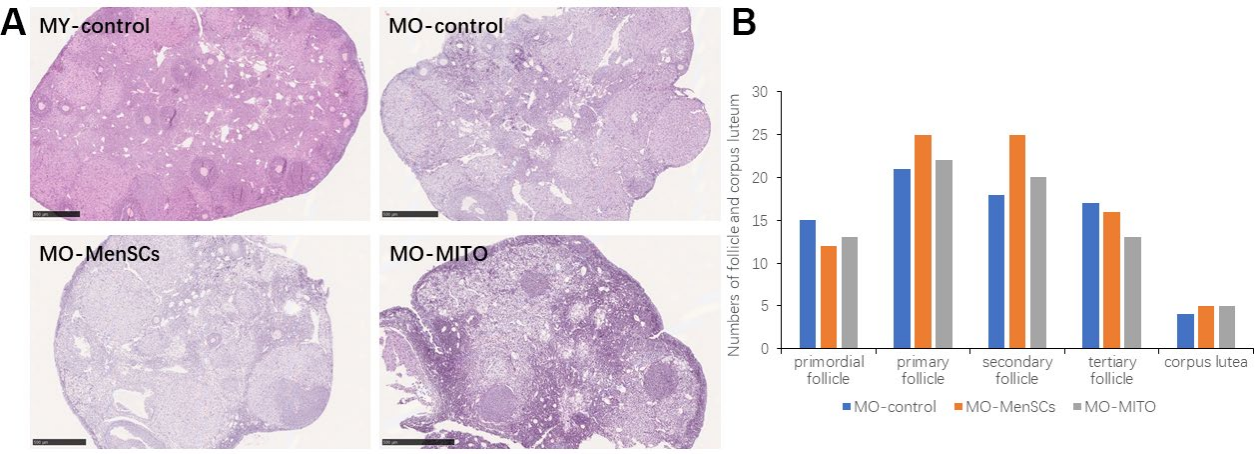
**MO-MenSCs and MO-MITO treatment groups showed similar effects on the aged ovary.** Representative images showing H&E-stained ovary sections in each group after seven days (**A**). Bar graph summarizing the number of growing follicles at various stages and corpus lutea in each group (**B**).

We further observed the histomorphology of the recipient ovaries by Hematoxylin-eosin (HE) staining. HE staining showed that the ovaries of 2–3 months young control group (MY-Control) were properly structured, with different stages of developing follicles, and layers of granulosa cells around the secondary follicle (Figure 2A). The aged control mice (MO-Control) showed disordered ovarian structure, with fewer number of growing follicles at each stage and granulosa cells as well (Figure 2A). MO-control ovaries had more atretic follicles, and enlarged intercellular space. MO-MenSCs and MO-MITO treatment moderately improved the morphology of aged ovaries (Figure 2A). The number of growing follicles at each stage and corpus lutea were counted in the MO-control, MO-MenSCs, and MO-MITO treatment groups. MO-MenSCs and MO-MITO treatment groups showed moderately higher number of primary and secondary follicles compared to the MO-control group (Figure 2B).

### Tracking donor MenSCs in the ovaries of recipient mice

**Figure 3:**
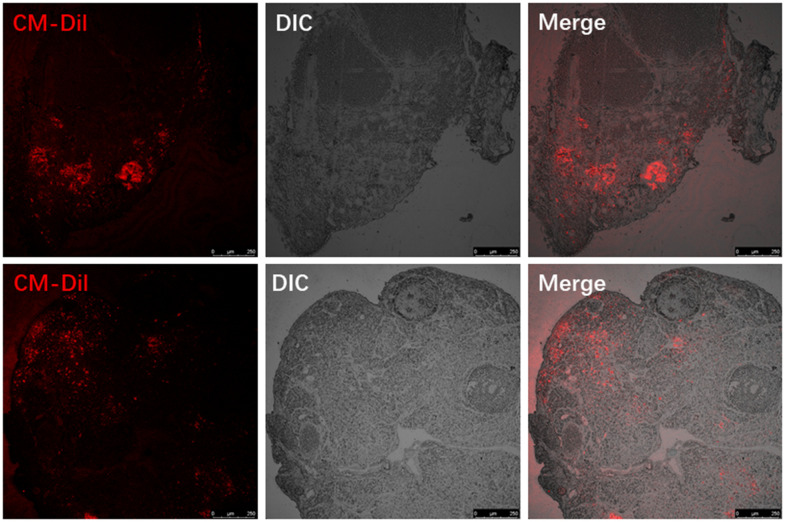
Visualizing the red fluorescent CM-Dil labelled MenSCs in the ovaries of two individual recipient mice.

We labelled donor MenSCs with red fluorescent CM-Dil and tracked them in the recipient mouse ovaries 7 days after treatment with MenSCs 3D gel. The ovaries from recipient mice were harvested after sacrifice, and paraffin sections were prepared to visualize the location of labelled MenSCs. CM-Dil red labelled MenSCs were clearly visible inside the recipient ovaries 7 days after treatment with 3D MenSCs gel (Figure 3). This result indicates that MenSCs can migrate into aged ovaries *in vivo*, which may repair the injury and improve the ovarian condition in aged mice.

### RNA expression analysis and related gene functions and pathways

**Figure 4:**
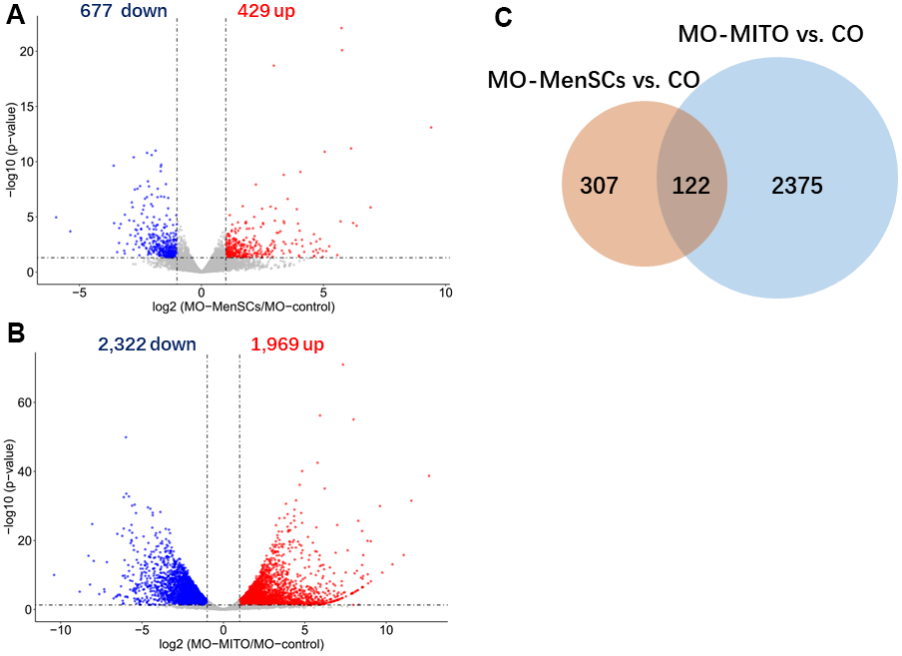
**Identification differently expressed genes by RNA-seq analysis.** Volcano plots showing differently expressed genes between MO-MenSCs vs. MO-control (**A**) and MO-MITO vs. MO-control (**B**) samples identified using transcriptomic data at day 7. Red dots denote the genes passing our p value and fold difference thresholds. The genes gene names of significant genes are marked. Venn diagram depicting the overlap of up-regulated genes between MO-MenSCs vs. MO-control and MO-MITO vs. MO-control comparisons (**C**).

**Figure 5:**
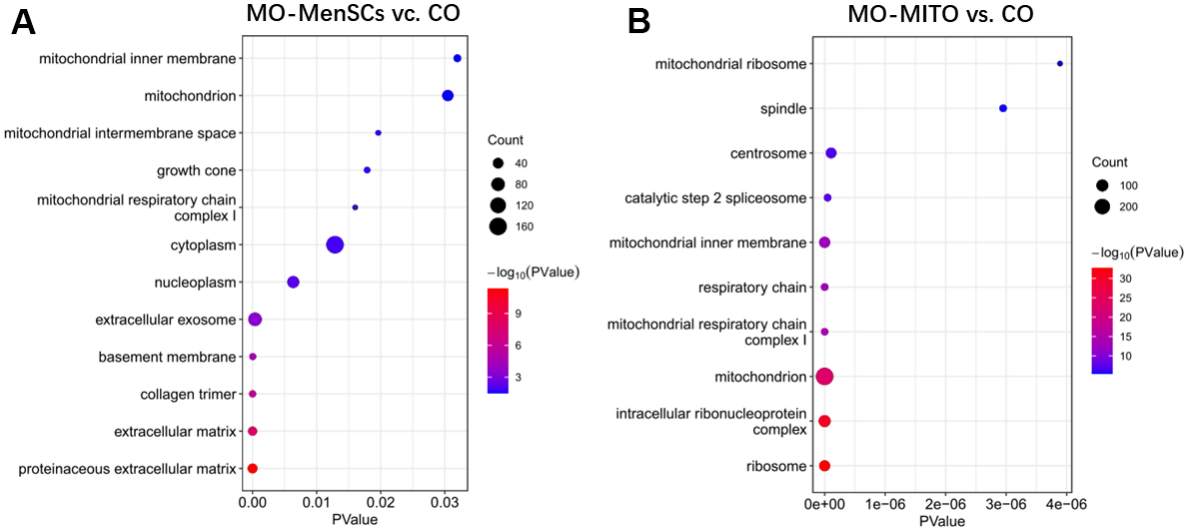
Functional annotation of up-regulated genes between MO-MenSCs vs. control (**A**) and MO-MITO vs. control samples (**B**).

Furthermore, to link ovary morphology changes and altered gene expression, RNA sequencing (RNA-seq) was performed to analyze the global transcriptomic profiles from MO-control and MO-MenSCs and MO-MITO treated ovaries. A sizable number of genes (429 upregulated and 677 downregulated) showed at least two-fold differential expression between MO-MenSCs and MO-control samples (Figure 4A). These differentially upregulated expressed genes were significantly enriched in cellular component (CC) categories related to proteinaceous extracellular matrix, extracellular matrix, collagen trimer, basement membrane, extracellular exosome, nucleoplasm, cytoplasm, mitochondrial respiratory chain complex I, growth cone, mitochondrial intermembrane space, mitochondrion, and mitochondrial inner membrane (Figure 5A).

Similarly, a total of 1,969 genes were upregulated and 2,322 genes were downregulated with at least two-fold change in MO-MITO samples compared to MO-control samples (Figure 4B). Of the genes that were upregulated in MO-MenSCs group, nearly 30% of them were also upregulated in MO-MITO group (Figure 4C). Significant enrichment of these 1,969 upregulated genes was observed in the following cellular components: ribosome, intracellular ribonucleoprotein complex, mitochondrion, mitochondrial respiratory chain complex I, respiratory chain, mitochondrial inner membrane, catalytic step 2 spliceosome, centrosome, spindle, and mitochondrial ribosome (Figure 5B). Interestingly the differentially expressed genes in both treatments were significantly enriched in mitochondria and their components (Figure 5A, 5B). This suggests that treatment of aged ovaries with MenSCs or their mitochondria may affect mitochondria related pathways.

**Figure 6:**
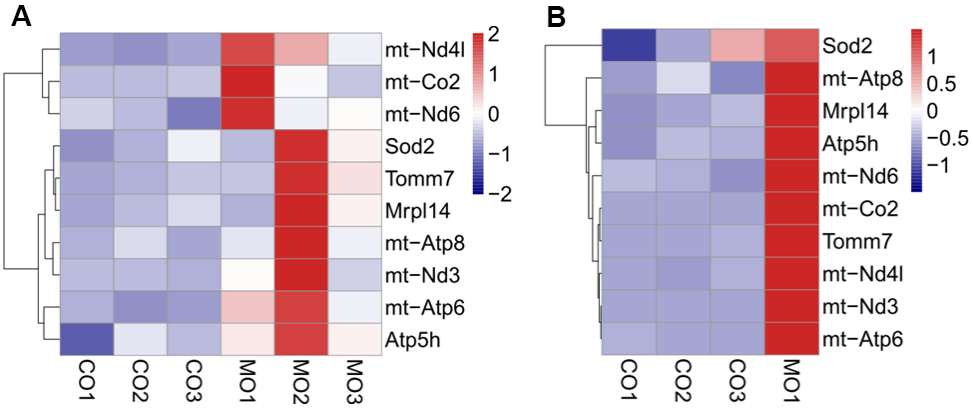
Heatmap showing the differential expression of mitochondrial related genes between MO-MenSCs vs. MO-control (**A**) and MO-MITO vs. MO-control samples (**B**).

We also found that several genes involved in mitochondrial pathways were upregulated upon MO-MenSCs or MO-MITO treatment in aged ovaries. Genes involved in mitochondrial ATP synthesis such as mt-Atp8, mt-Co2, mt-Nd3, mt-Nd4l, Got2-ps1, Tomm7, mt-Atp6, mt-Tl2, Atp5k, mt-Te, and NADH dehydrogenase were significantly upregulated in the MO-MenSCs treatment group compared to MO-control group (Figure 6A). Similarly, in the MO-MITO treatment group, mt-Nd1, Immp2l, Mrpl39, mt-Ti, Mrpl45, Slc25a29, Slc25a14, Mrpl48-ps, Ucp3, Mrps17, Tomm20 and other mitochondrial-related genes were upregulated, whereas the autophagy pathway molecule Prkn was significantly down-regulated compared to the MO-control group (Figure 6).

## DISCUSSION

Female fertility decreases significantly after 37 years and begins to fade after 45 years of age in humans. Histological analysis of the ovaries of 40–48 weeks old mice showed significantly reduced number of follicles at different stages, and increased number of atretic follicles. These results suggest that 40–48 weeks (10–12 months) old mice could represent women of age over 38 years old and can be used to study ovarian aging.

Mitochondria play a significant role in the complex process of oocyte development. In addition to supplying energy for granulosa cell proliferation and oocyte development, mitochondria can also regulate important physiological processes such as antioxidant defense and apoptosis of granulosa cells. Additionally, mitochondria maintain the structural integrity of granulosa cells, their function, and prevent the damage caused by mutations. A study on mouse oocytes revealed an age-associated alteration of gene expression patterns including the genes involved in mitochondrial functions and oxidative stress [20]. The quality of oocytes is a key factor affecting the outcome of pregnancy. The quality of oocytes declines with ovarian ageing, leading to a decline in female fertility. Our transcriptomic analysis revealed that treatment with MenSCs upregulated the expression of several mitochondrial genes involved in ATP synthesis, along with NADH dehydrogenase indicating that they promote mitochondrial ATP synthesis in aging ovary. It is known that insufficiency in cell energetics due to low mitochondrial ATP synthesis could cause chromosomal aberrations and could affect oocyte number and embryo health. Aging is associated with reduced mitochondrial function in granulosa cells [21]. Interestingly, ATP6 and ATP8 genes encoding two subunits of the ATP synthase complex (complex V of the respiratory chain) itself, were among the ovarian genes that were upregulated upon treatment with MenSCs. Thus, MenSCs could restore the function of aging ovaries and improve oocyte quality by promoting mitochondrial ATP synthesis which is reflected in our RNA-seq data [22–25].

To summarize, we treated the ovaries of aged mice with MenSCs or their mitochondria, performed morphological analysis of the treated ovaries, and analyzed the changes in the ovarian transcriptome. Treatment with MenSCs or their mitochondria moderately improved the morphology and function of aged ovaries. However, several genes involved in mitochondrial function, particularly ATP biosynthesis were upregulated in aged ovaries treated with MenSCs or their mitochondria. We speculate that the treatment of aged ovaries aged by MenSCs or their mitochondria may restore ovarian function by promoting mitochondrial function, thereby providing a potential therapeutic strategy for improving ovarian reserve and treatment of infertility in elderly women.

## MATERIALS AND METHODS

### Major reagents

The major reagents and antibodies used in this study were Collagenase I (Sigma, USA), DMEM (GIBCO, USA), 0.25% Trypsin-EDTA (GIBCO, USA), FITC Anti-CD34 (Ebioscience, USA), FITC Anti-CD45 (Ebioscience, USA), PE Anti-CD29 (Ebioscience, USA), FITC Anti-CD90 (Ebioscience, USA), PE Anti-CD73 (Ebioscience, USA).

### Animals

Experiments involving animals were approved by the General Hospital Animal Ethics Committee of PLA and were performed in accordance with the NIH Guidelines on the Care and Use of Laboratory Animals. Female CD-1 (ICR) mice aged 10–12 months were used as donors and recipients in the experiment.

### Isolation of MenSCs

For preparation of MenSCs, 10–12 months CD-1 (ICR) female mice were sacrificed for cervical dissection, and the bilateral uterus was isolated under aseptic conditions. The fat tissue and blood vessels surrounding the uterine horn were removed and the uterus washed with PBS. The uterus was then cut longitudinally along the uterine cavity to expose the endometrium under microscope, and the endometrium was carefully isolated at 4° C. The tissues were minced and digested for 30–60 minutes with gentle agitation in serum-free medium using 0.1% type I collagenase (Sigma, USA). Then, the enzyme was inactivated and a 200-μm mesh filter was used to the filter samples. The filtered samples were centrifuged at 1200rpm for 5 minutes to pellet the cells. The isolated cells were cultured in DEME low-glucose medium with 10% FBS, 50 U/mL penicillin, and 50 μg/mL streptomycin in a humidified incubator at 37° C with 5% CO_2_ [26]. To ensure the purity of the MenSCs, we identified MenSCs by flow cytometry (Supplementary Figure 1).

### Isolation of mitochondria

Mitochondria were isolated from MenSCs by differential centrifugation. The MenSCs were suspended in PBS at 10^6^ cells per mL. Then the cells were homogenized 4–6 times on ice using a cell homogenizer. The homogenate was then centrifuged for 20 min at 2000 rpm, the supernatant was collected and again centrifuged for 20 min at 10000 rpm for the mitochondria to sediment [26]. All the processes were carried out at 4° C, and the extracts were maintained at 4° C until use.

### Preparation of MenSCs 3D alginate gel and mitochondrial 3D alginate gel

The preparation alginate gel was performed as described previously [27, 28]. Sodium alginate powder (Sigma-Aldrich, USA) was dissolved in PBS to form 2% sodium alginate solution at room temperature. The MenSCs were digested with 0.25% trypsin and added into the alginate solution, the cell concentration was adjusted to 10^6^ cells per mL. Then, the cell suspension droplets were added into 1% calcium chloride solution to form a 3D stem cell gelatinous substance. Similarly, we extract mitochondria from 10^6^ stem cells to prepare 3D mitochondria gel. The whole encapsulation process is carried out under aseptic conditions.

### Preparation of fluorescent labeled MenSCs

Cultured MenSCs were incubated with 4 μL/mL of CM-Dil and incubated for 30 min at 37° C, shaken once every 10 minutes. After incubation, the cells were washed twice with PBS to remove the excess dye [29, 30].

### Treatment of MenSCs gel and MITO gel to the ovaries of recipient mice

Recipient mice (10–12 months old) were individually anesthetized with 0.2 mL of pentobarbital (0.2%). After the mice were anesthetized, they were placed flat on their abdomen, fixed and the dorsal side of the abdomen was disinfected with 75% alcohol. Incision was made to cut the skin and muscle to expose the ovaries. 20–50 μL of control gel, MenSCs gel, or mitochondrial gel was applied it around both ovaries After surgery, the mice were injected with 100,000 U of penicillin to prevent infection. Three recipient mice were used in each of the control and MO-MenSCs and MO-MITO treatment groups.

### Morphological examination of mouse ovaries

Seven days after treatment with the control or MenSCs or MITO 3D gel, both ovaries of the recipient mice were isolated and fixed in 4% paraformaldehyde overnight. Following fixation, the ovaries were dehydrated and embedded in paraffin blocks and sections were immobilized on slides. Ovarian sections were stained with hematoxylin and eosin. The CM-Dil red fluorescence of mesenchymal stem cells in the treated ovaries were also imaged within the paraffin section under a fluorescence microscope.

### RNA library preparation and sequencing

Ovary samples were homogenized in TRIzol (Invitrogen) and processed according to the manufacturer’s instructions. 2 ug of total RNA from each sample was processed using the NEBNext®Ultra II kit (New England Biolabs, Inc.) according to the manufacturer's instructions. Briefly, mRNAs were captured with oligo-dT magnetic beads and fragmented for 6 min at 94° C. First strand synthesis was performed on fragmented RNA, followed by second strand synthesis and dA-tailing. Adapter ligation was performed using KAPA single-indexed adapters (KAPA, Pleasanton, CA). The adapter-ligated fragments were amplified with 10 PCR cycles. Final libraries were then pooled and sequenced on a HiSeq4000 single-end 50 nt system (Illumina).

### RNA-seq data analysis

FASTQ sequence files from biological replicates were concatenated and aligned to the M. musculus reference genome (mm10) using Hisat2 [31]. FeatureCounts software was used for counting reads to each exon or each gene for read summarization [32]. Differentially expressed genes with two-fold or higher change, and p < 0.05 (unpaired t-test) were identified using the DESeq2 program [33]. Enrichment analysis of differentially expressed genes was performed using the web-based software DAVID and including the cellular component Gene Ontology vocabulary. Statistical parameters to consider significant enrichment were p < 0.01, and fold enrichment > 2.

## Supplementary Material

Supplementary Figure 1
