# Correction for: Menstrual blood-derived stem cells and its mitochondrial treatment improve the ovarian condition of aged mice

**DOI:** 10.18632/aging.204186

**Published:** 2022-07-15

**Authors:** Qi Zhang, Chunlei Liu, Ling Yu, Xiaona Wang, Jianxiu Hao

**Affiliations:** 1 Medical School of Chinese PLA, Department of Obstetrics and Gynecology, The First Medical Center of PLA General Hospital, Beijing, 100853, China; 2 Department of Transformation Medicine Center, The Medical Innovation Research Division of Chinese PLA General Hospital, Beijing, 100853, China; 3 Senior Department of Obstetrics and Gynecology, The Seventh Medical Center of PLA General Hospital, Beijing, 100853, China; 4 Key Laboratory of RNA Biology, Center for Big Data Research in Health, Institute of Biophysics, Chinese Academy of Sciences, Beijing, 100101, China; 5 Department of Clinical Biobank Center, The Medical Innovation Research Division of PLA General Hospital, Beijing, 100853, China

**This article has been corrected:** The authors corrected the FUNDING section. The correct section is presented below.

