# Supplementary material for: Menstrual blood-derived stem cells and its mitochondrial treatment improve the ovarian condition of aged mice
Source: Aging (Albany NY). 2022 May 3;14(9):3826–35. doi: 10.18632/aging.204043 (PMC9134964; doi:10.18632/aging.204043)
Supplement: Supplementary Figure 1 [file aging-14-204043-s001.pdf]

SUPPLEMENTARY FIGURE

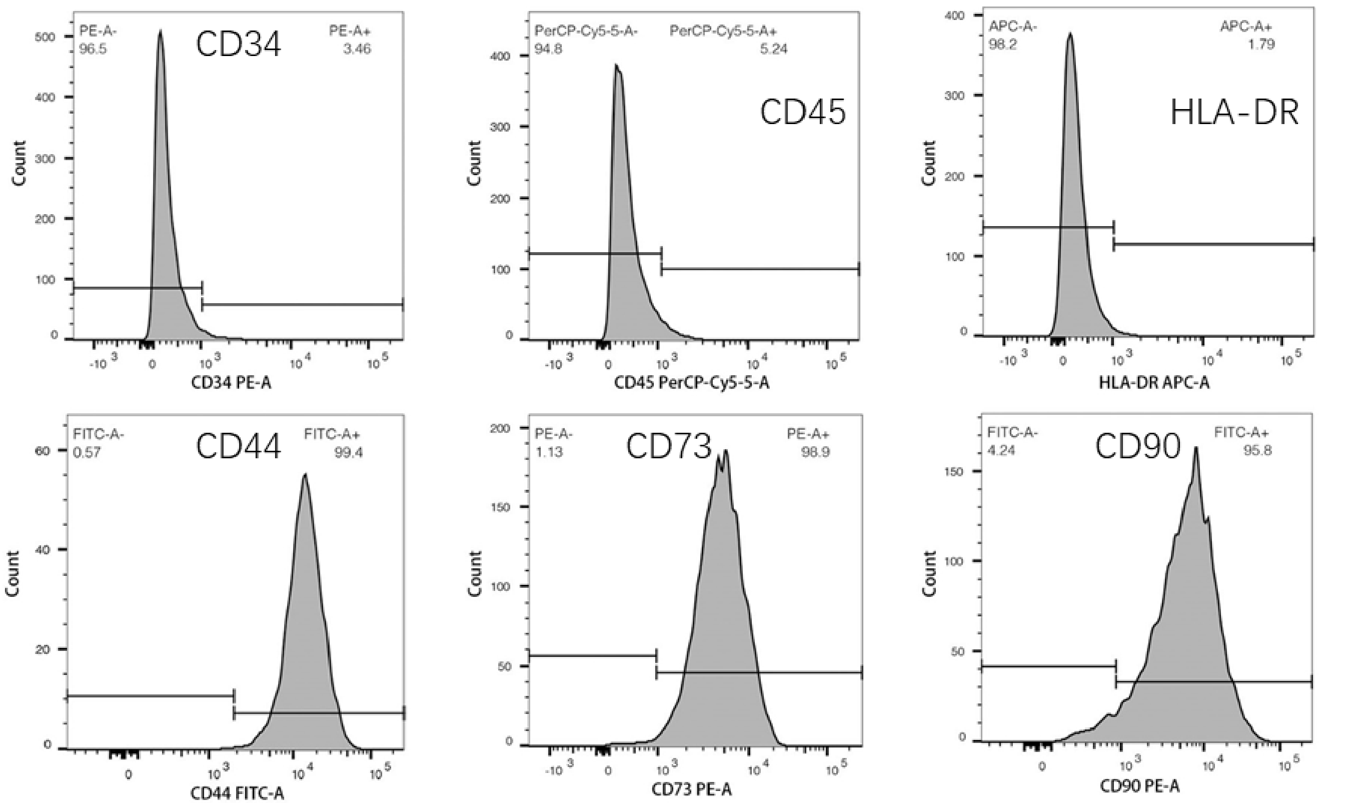

Supplementary Figure 1. Flow-cytometric detection of surface markers of fourth-generation MenSCs. All horizontal coordinates in the figure indicate fluorescence intensity, and all vertical coordinates indicate cell counts.
